# The Diagnostic Performance of Maximum Uptake Value and Apparent Diffusion Coefficient in Differentiating Benign and Malignant Ovarian or Adnexal Masses: A Meta-Analysis

**DOI:** 10.3389/fonc.2022.840433

**Published:** 2022-02-09

**Authors:** Xianwen Hu, Zhigang Liang, Chuanqin Zhang, Guanlian Wang, Jiong Cai, Pan Wang

**Affiliations:** ^1^ Department of Nuclear Medicine, Affiliated Hospital of Zunyi Medical University, Zunyi, China; ^2^ Research and Development Department, Jiangsu Yuanben Biotechnology Co., Ltd., Zunyi, China

**Keywords:** ovarian cancer, maximum uptake value, apparent diffusion coefficient, PET/CT, diffusion weighted imaging

## Abstract

**Objective:**

The purpose of this meta-analysis was to provide evidence for using maximum uptake value (SUVmax) and apparent diffusion coefficient (ADC) to quantitatively differentiate benign and malignant ovarian or adnexal masses, and to indirectly compare their diagnostic performance.

**Material and Methods:**

The association between SUVmax, ADC and ovarian or adnexal benign and malignant masses was searched in PubMed, Cochrane Library, and Embase databases until October 1, 2021. Two authors independently extracted the data. Studies included in the analysis were required to provide data for the construction of a 2 × 2 contingency table to evaluate the diagnostic performance of SUVmax or ADC in differentiating benign and malignant ovarian or adnexal masses. The quality of the enrolled studies was evaluated by Quality Assessment of Diagnostic Accuracy Studies-2 (QUADAS-2) instrument, and the meta-analysis was conducted using Stata software version 14.0. Forest plots were generated according to the sensitivity and specificity of SUVmax and ADC, and meta-regression analysis was further used to assess heterogeneity between studies.

**Results:**

A total of 14 studies were finally included in this meta-analysis by gradually excluding duplicate literatures, conference abstracts, guidelines, reviews, case reports, animal studies and so on. The pooled sensitivity and specificity of SUVmax for quantitative differentiation of benign and malignant ovarian or adnexal masses were 0.88 and 0.89, respectively, and the pooled sensitivity and specificity for ADC were 0.87 and 0.80, respectively.

**Conclusion:**

Quantitative SUVmax and ADC values have good diagnostic performance in differentiating benign and malignant ovarian or adnexal masses, and SUVmax has higher accuracy than ADC. Future prospective studies with large sample sizes are needed for the analysis of the role of SUVmax and ADC in the differentiation of benign and malignant ovarian or adnexal masses.

## Introduction

Ovarian cancer is one of the most common and deadly malignant tumors of the female reproductive tract. According to the latest statistics, 314,000 new ovarian cancer patients and 207,000 new deaths were due to ovarian cancer in the world in 2020, seriously threatening the lives and health of women (1). Ovarian neoplasms have a variety of morphological characteristics and genetic and epigenetic changes, and lack of specific clinical manifestations, so it is challenging to distinguish benign from malignant ovarian lesions (2). Approximately 60% of ovarian cancer patients are already at an advanced stage when diagnosed, resulting in a five-year survival rate of only about 27% for these patients (3, 4). Therefore, it is of great significance to find a highly sensitive and specific diagnostic method for correct diagnosis, clinical management, and prognosis evaluation of ovarian cancer.

Imaging examination and serum tumor marker examination are common screening methods for gynecological diseases. However, the increase of serum tumor markers is not specific for the diagnosis of ovarian cancer, and can also be positive in some benign diseases such as pelvic inflammatory disease, ovarian cyst, and endometriosis (5). Ultrasound is the most commonly used imaging method for ovarian or adnexal lesions, but it has some limitations due to low resolution, obesity, and interference of intestinal gas artifacts (6). Computed tomography (CT) and magnetic resonance imaging (MRI) can provide anatomic information of ovarian lesions and surrounding tissues, which is of great clinical significance to determine the nature, extent of involvement, and treatment decision of ovarian lesions. Compared with CT, MRI has a higher soft tissue resolution. As a sequence of MRI, diffusion weighted imaging (DWI) has been proved to be of high diagnostic value in differentiating benign and malignant adnexal masses, especially entirely solid non-fatty, non-hemorrhagic masses, or complex masses that are either septated cysts or combined solid and cystic masses (7). However, DWI still presents a great challenge in differentiating mature teratoma from benign tumor and ovarian fibroma from malignant tumor (8). Positron emission tomography (PET)/CT imaging integrates CT and PET into one machine, simultaneously realizing the combination of anatomical imaging and functional imaging, which can intuitively reflect the changes of tumor cell metabolism and make accurate diagnosis in the early stage of tumor. Previous meta-analyses have shown that PET/CT has a high accuracy in differentiating ovarian or adnexal benign and malignant tumors (9). However, the quality of the above study was limited by the different methods used in the included studies and the incomplete use of quantitative data for differentiating ovarian benign and malignant tumors. In order to solve this problem, this study conducted a meta-analysis based on published high-quality studies to quantitatively evaluate the diagnostic performance of maximum uptake value (SUVmax) of ^18^F-FDG PET/CT and apparent diffusion coefficient (ADC) values of DWI-MRI in differentiating benign and malignant ovarian tumors.

## Materials and Methods

### Study Search Strategy

The present meta-analysis was performed in accordance with the 2009 PRISMA guidelines (10). The Pubmed, Cochrane Library, and Embase databases were searched for articles reporting on SUVmax or ADC values in the differentiation of ovarian or adnexal masses and included in the studies. The search terms for a complete search strategy were as follows: (“PET/CT” OR “PET-CT” OR “positron emission tomography/computed tomography” OR “positron emission tomography-computed tomography” OR “SUVmax” OR “Maximum standard uptake value” OR “MR” OR “Magnetic Resonance” OR “diffusion weighted imaging” OR “diffusion magnetic resonance imaging” OR “apparent diffusion coefficient” OR “ADC”) AND (“ovarian neoplasms” OR “ovary neoplasms” OR “ovary cancer” OR “ovarian cancer” OR “cancer of ovary” OR “ovarian tumor” OR “ovarian carcinoma” OR “adnexal mass” OR “adnexal lesions”). Original studies published in English with a deadline of October 1, 2021 were included. Articles were screened by two reviewers with 10 years of meta-analysis writing experience. All articles that might be suitable for this study were reserved after reading abstracts. In case of disputes, the team members discussed them until a consensus was reached.

### Study Selection and Exclusion Criteria

The published studies included in the current meta-analysis are required to meet the following inclusion criteria: i) articles whose full text is in English before the deadline for publication on October 1, 2021; ii) prospective or retrospective study of accuracy in differentiating benign and malignant ovarian or adjunctive masses using SUVmax of ^18^F-FDG PET/CT and/or ADC values of DWI-MRI; iii) studies can directly extract or calculate the true positive, false positive, false negative, and true negative values based on the sensitivity, specificity, negative predictive value, positive predictive value in the article to construct a 2 × 2 contingency table; and iv) interpretation of the results should include at least histopathological findings. The exclusion criteria included: i) studies with sample sizes less than 30 patients; ii) although studies used SUVmax or/and ADC values to differentiate benign and malignant ovarian or adnexal masses, the cut-off value was not described in detail; iii) studies are published duplicately or contain overlapping data; iv) for studies of ADC values differentiating benign from malignant ovarian or adjunctive masses, magnetic field intensity of MRI should be described and studies of less than 1.5 Tesla (T) should be excluded; and v) for SUVmax, studies using tracers other than ^18^F-FDG.

### Data Extraction and Quality Assessment

A standard data extraction form were used from enrolled studies, and descriptive information on a range of factors was independently collected, namely, the first author, publication year, country, study design type (retrospective or prospective), number of patients, mean age, patient selection (consecutive or nonconsecutive), positive reference standard, the cutoff value of SUVmax for PET/CT, and ADC value for DWI-MRI, the interval time between index tests and HP, true-positive (TP), false-positive (FP), false-negative (FN), and true-negative (TN) results from the included studies. In addition, data were extracted on the characteristics and techniques of the scanners used in the study, such as magnetic field strength for MRI, CT technology (whether contrast agent contrast enhancement scan is used) for PET/CT and so on. The Quality Assessment of Diagnostic Accuracy Studies-2 (QUADAS-2) was used to evaluate the quality of the enrolled studies (11). Data extraction and critical evaluation of article quality are conducted independently by two authors, and they will negotiate together until a consensus was reached in case of disputes.

### Statistical Analyses

The current meta-analysis was conducted using Stata software (version 14.0; Stata Corporation, College Station, TX, USA). The specificity, sensitivity, positive likelihood ratio (PLR), negative likelihood ratio (NLR), diagnostic odds ratio (DOR) and summary receiver operating characteristic (SROC) curve to count the area under the curve (AUC) with their 95% confidence intervals (CIs) calculated based on the TP, FP, FN, and TN values extracted from the enrolled studies were used to evaluate the diagnostic performance of SUVmax and ADC value in differentiating between benign and malignant ovarian or adnexal masses. The general estimates of sensitivity and specificity of enrolled studies were calculated using hierarchical logistic regression models, namely, hierarchical summary receiver operating characteristics (HSROC) models and concomitant variables. The HSROC curve with a 95% confidence and prediction region is used to plot its sensitivity and specificity results. Heterogeneity between studies was assessed using Cochran’s Q test and Higgins I^2^ test (12). For Cochran’s Q test, P <0.05 was the test standard, indicating that there was heterogeneity among the enrolled studies. The criteria for evaluating the degree of heterogeneity using Higgins I^2^ test is: inconsistency index (I^2^) <50% was deemed as irrelevant heterogeneity; I^2^ = 50–80% was considered to be moderate heterogeneity; I^2^ >80% indicated significant heterogeneity. The funnel plots and Deeks’ asymmetry tests were used as the assessment of publication bias for ADC value of DWI-MRI and SUVmax of ^18^F-FDG PET/CT (13). A two-sample Z-test was conducted to evaluate the difference in diagnostic performance between the two diagnostic methods, and p <0.05 was considered to be statistical significance in the diagnostic performance of the two methods in the quantitative differentiation of benign and malignant ovarian or adnexal masses.

## Results

### Literature Search

**Figure 1:**
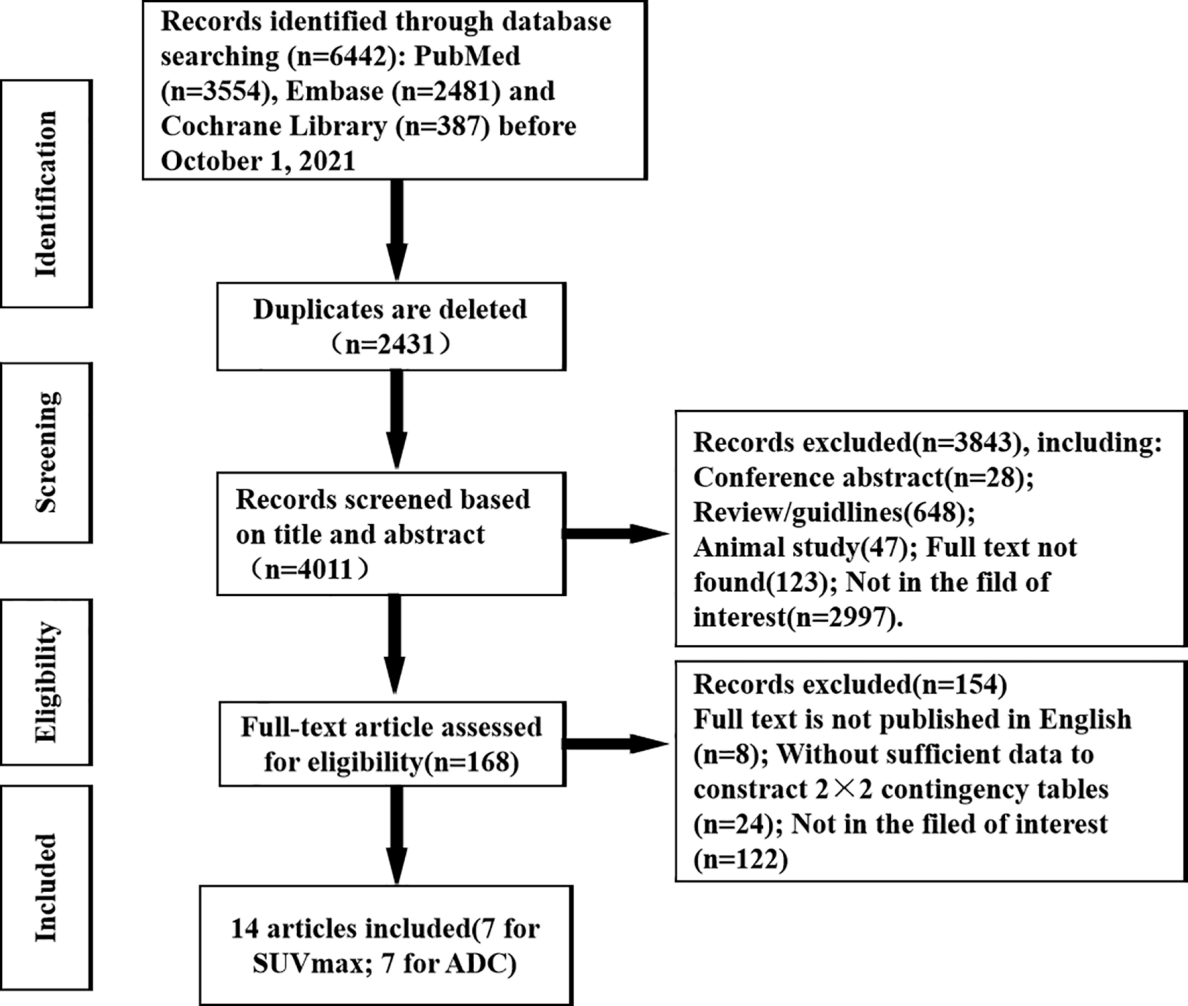
Flow chart of the research selection process.

A total of 6,302 articles were retrieved from three databases by subject terms, consisting of 3,554 articles in PubMed, 2,361 articles in Embase and 387 articles in Cochrane Library. After gradually ruling out overlapping, irrelevant comments, guidelines, conferences, case studies, animal studies, and articles not in the field of interest, etc., 6,174 articles were excluded, and the remaining 128 potentially eligible original texts were further evaluated. By carefully reading the full text, 116 articles were further excluded, including studies that not the full text was published in English, data could not be extracted to construct a contingency table, and papers were in areas of non-interest. Finally, a total of 14 articles, namely, 7 using SUVmax of ^18^F-FDG PET/CT and 7 using ADC values of DWI-MRI to quantitatively differentiate the diagnostic performance of benign and malignant ovarian or adnexal masses were included in this meta-analysis (14–27). The detailed retrieval process of the literature is shown in **Figure 1**.

### Study Characteristics

The 14 enrolled studies included a total of 1,317 patients with 1,373 masses, consisting of 3 prospective studies, 10 retrospective studies, and 1 was unspecified. The enrolled studies were published from 2007 to 2020, with sample sizes ranging from 30 to 191 and mean ages of patients ranging from 39.3 to 64. At least histopathological results were used as a reference for positive interpretation in all of the enrolled studies, and follow-up results were also used in two of the studies. Follow-up included imaging findings and serum tumor markers for at least six months. For SUVmax of ^18^F-FDG PET/CT, two studies used both non-contrast-enhanced scanning and contrast-enhanced CT technique (16, 19). As for ADC, five studies used magnetic field strengths of 1.5 (T), one of 3.0 T and one of both. The detailed characteristics of the enrolled studies are summarized in **Table 1**. The characteristics of the enrolled studies to differentiate benign and malignant ovarian or adnexal masses by quantitative SUVmax of PET/CT are shown in **Table S1**, and the characteristics for quantitative ADC values of DWI-MRI are shown in **Table S2**.

**Table 1:** The main characteristics of the enrolled studies.

Study/Year/Country	No. of patients	Mean age	Study design	Consecutive	Technique		Cutoff value	Reference standard	interval time	Mean value	
SUVmax										benign	malignant
Castellucci/2007/Italy (14)	50	64	P	Yes	PET/CT (non-CE)		3.0	HP + follow-up >6 months	≤2W	NR	NR
Yamamoto/2008/Japan (15)	30	47.7	P	Yes	PET/CT (non-CE)		3.0	HP	NR	1.74	9.32
Kitajima/2011/Japan (16)	108 (111 tumors)	55.4	NR	NR	PET/CT (non-CE and CE)		2.55	HP	NR	2.0	7.55
Zytoon/2012/Egypt (17)	98	57.7	P	yes	PET/CT (non-CE)		4.3	HP + follow-up (imaging + tumor marker)	≤4W	NR	NR
Tanizaki/2014/Japan (18)	160	NR	R	NR	PET/CT (non-CE)		2.9	HP	NR	NR	NR
Lee/2015/Korea (19)	39	51	R	NR	PET/CT (non-CE and CE)		2.5	HP	≤7W	2.4	10.5
Takagi/2018/Japan (20)	76	59	R	NR	PET/CT (non-CE)		3.97	HP	NR	2.48	10.98
ADC						*b* value	(10^-3^s/mm^2^)			10^-3^s/mm^2^	
Li/2012/China (21)	127 (131 tumors)	59.9	R	Yes	MRI (1.5T)	0,1000	1.25	HP	NR	1.69	1.03
Zhang/2012/China (22)	191 (202 tumors)	56.5	R	Yes	MRI (1.5T)	0,1000	1.2	HP	NR	1.22	0.91
Fan/2015/China (23)	64	46.7	R	NR	MRI (3.0T)	0,1000	0.878	HP	NR	1.325	0.878
Zhang/2019/China (24)	85	52.7	R	Yes	MRI (1.5T)	0,800	1.162	HP	NR	NR	NR
Türkoglu/2020/Turkey (25)	43	51.26	R	Yes	MRI (1.5T)	0,800	0.93	HP	≤1W	1.37	0.92
Mansour/2015/Egypt (26)	197 (235tumors)	39.3	R	Yes	MRI (1.5T)	0,1000	1.2	HP	NR	1.2	0.83
Takeuchi/2010/Japan (27)	49	59	R	Yes	MRI (1.5/3.0T)	0,800	1.15	HP	NR	1.38	1.03

HP, Histopathology; P, Prospective; R, Retrospective; CE, Contrast enhancement; non-CE, none contrast enhancement; NR, Not report; W, Week; MRI, Magnetic resonance imaging; PET/CT, Positron emission computer/Computed tomography; T, Tesla; SUVmax, Maximum uptake value; ADC, Apparent diffusion coefficient.

### Quality Evaluation

The quality of all 14 enrolled studies met at least five of the 7 reference criteria (namely, four items in the risk of bias, patient selection, index test, reference standard, flow and timing and three in application concerns, patient selection, index test, reference standard) was therefore considered satisfactory. For the risk of reference standard bias, all studies at least used histopathological findings as a positive interpretation and were considered low risk. Regarding the risk of bias for flow and time, 10 of the 14 studies did not report the time interval between the index and the reference standard test, so the risk of bias was unclear. All the patients in the enrolled studies were suspected of having ovarian or adnexal masses by ultrasound or serum tumor markers, so the risk of publication bias and application concerns in patient selection were considered low. The results of the QUADAS-2 assessment are shown in **Table 2**.

**Table 2:** Risk of bias and application concerns for included studies were assessed by the QUQUADAS-2 tool.

Study	Risk of bias				Application concerns		
	Patient selection	Index test	Reference standard	Flow and timing	Patient selection	Index test	Reference standard
Castellucci/2007 (14)	L	U	L	L	L	L	L
Yamamoto/2008 (15)	L	L	L	U	L	L	L
Kitajima/2011 (16)	L	L	L	U	L	L	L
Zytoon/2012 (17)	L	L	L	L	L	L	L
Tanizaki/2014 (18)	L	L	L	U	L	L	L
Lee/2015 (19)	L	U	L	L	L	L	L
Takagi/2018 (20)	L	L	L	U	L	U	L
Li/2011 (21)	L	L	L	U	L	L	L
Zhang/2012 (22)	L	L	L	U	L	L	L
Fan/2015 (23)	L	L	L	U	L	L	L
Zhang/2019 (24)	L	L	L	U	L	L	L
Türkoğlu/2020 (25)	L	U	L	L	L	L	L
Mansour/2015 (26)	L	L	L	U	L	U	L
Takeuchi/2010 (27)	L	L	L	U	L	L	L

L, low; U, unclear.

### Diagnostic Accuracy

**Figure 2:**
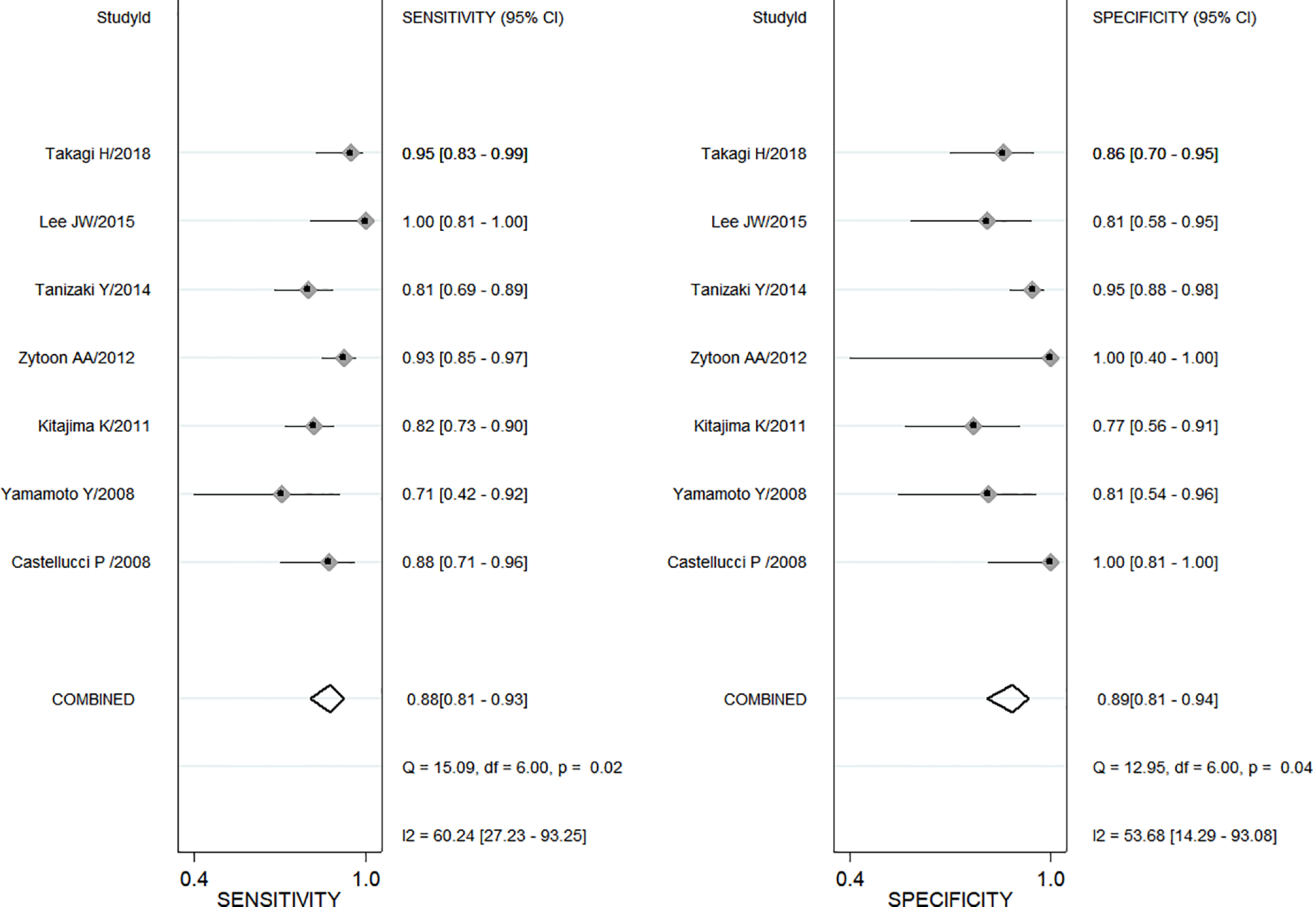
The pooled sensitivity and specificity for maximum uptake value (SUVmax).

A total of seven enrolled studies using SUVmax to differentiate ovarian or adnexal benign and malignant tumors had sensitivity ranging from 0.71 (95% CI, 0.42–0.92) to 1.0 (95% CI, 0.81–1.00) and specificity ranging from 0.77 (95% CI, 0.56–0.91) to 1.0 (95% CI, 0.81–1.00), with a pooled sensitivity and specificity of 0.88 (95% CI, 0.81–0.93) and 0.89 (95% CI, 0.81–0.94), respectively, as shown in **Figure 2**. Both Cochran’s Q test and Higgins I^2^ test showed moderate heterogeneity in sensitivity (Q = 15.02, p = 0.02; I^2^ = 60.24) and specificity (Q = 12.95, p = 0.04; I^2^ = 53.68).

**Figure 3:**
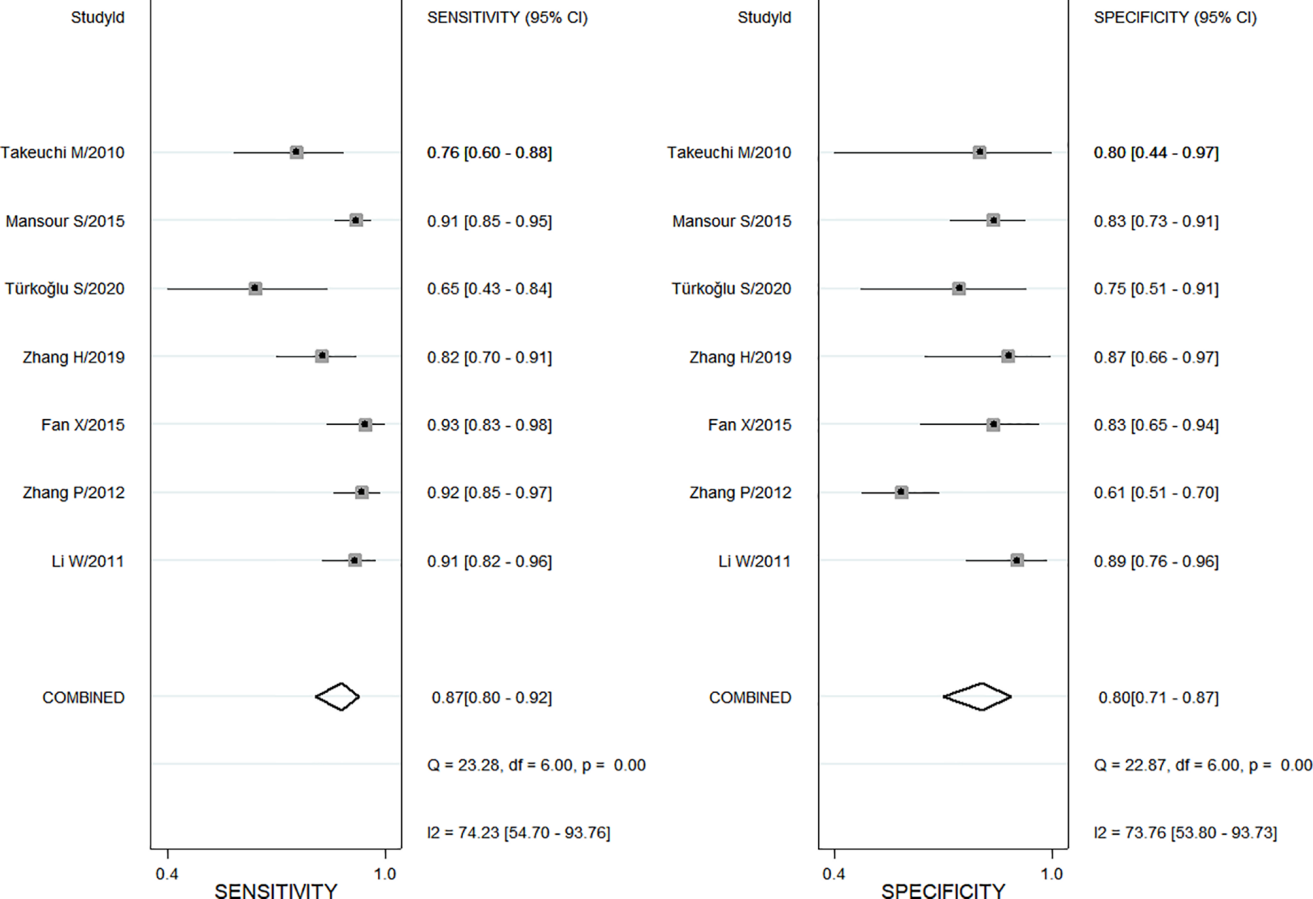
The pooled sensitivity and specificity for apparent diffusion coefficient (ADC).

Also, a total of 7 studies using ADC values of DWI-MRI evaluated the diagnostic performance of benign and malignant ovarian or adnexal masses, with sensitivity ranging from 0.65 (95% CI, 0.43–0.84) to 0.93 (95% CI, 0.83–0.98) and specificity ranging from 0.61(95% CI, 0.51–0.70) to 0.89 (95% CI, 0.76–0.96), for a pooled sensitivity and specificity of 0.87 (95% CI, 0.80–0.92) and 0.80 (95% CI, 0.71–0.87), respectively, as shown in **Figure 3**. Both Cochran’s Q test and Higgins I^2^ test showed heterogeneity among studies in sensitivity (Q = 23.28, *p* <0.01; I^2^ = 74.23) and specificity (Q = 22.87, *p* <0.01; I^2^ = 73.76), too.

**Figure 4:**
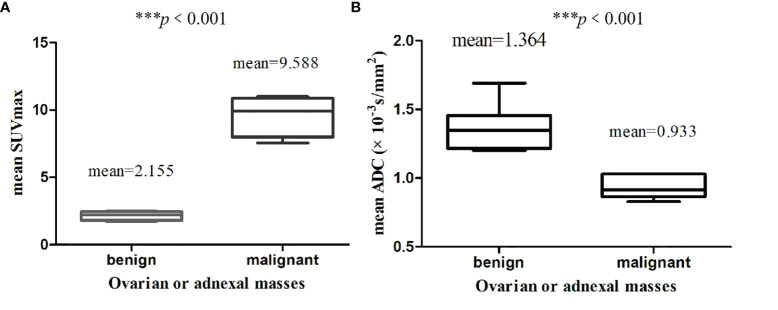
Comparison of SUVmax **(A)** and ADC values **(B)** between benign and malignant lesions.

**Figure 5:**
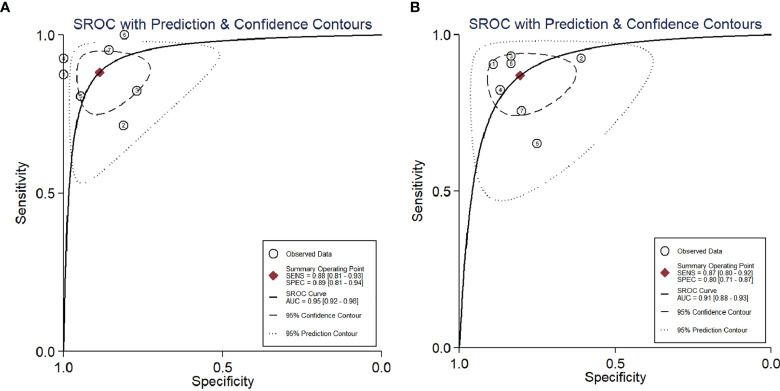
SROC curve of the diagnostic performance of SUVmax **(A)** and ADC **(B)** for ovarian cancer. AUC, area under the curve; SENS, sensitivity; SPEC, specificity; SROC, summary receiver operating characteristic.

The calculated mean SUVmax for benign and malignant ovarian or adnexal masses was 2.155 and 9.588, respectively, with significant statistical difference (*p* <0.001).The graphical distribution of SUVmax in benign and malignant ovarian or adnexal masses is shown in **Figure 4A**. The calculated mean ADC values for benign and malignant ovarian or adnexal masses were 1.364 × 10^−3^ mm^2^/s and 0.933 × 10^−3^ mm^2^/s, respectively, also showing significant statistical differences (**Figure 4B**). The PLR of SUVmax of ^18^F-FDG PET/CT and ADC of DWI-MRI were 7.8 (95% CI, 4.5–13.6) and 4.4 (95% CI, 2.9–6.8), and the NLR was 0.13 (95% CI, 0.08–0.21) and 0.16 (95% CI, 0.11–0.25), respectively. The pooled DOR for benign and malignant ovarian or adnexal masses assessed by SUVmax and ADC was 59 (95% CI, 27–128) and 27 (95% CI, 14–54), with a pooled AUC of 0.95 (95% CI, 0.92–0.96) and 0.91 (95% CI, 0.88–0.93), respectively, as shown in **Table 3**. Overall, there was no statistical difference in sensitivity and specificity of using SUVmax and ADC values in the assessment of benign and malignant ovarian or adnexal masses, with *p*-values of 0.705 and 0.166, respectively. The area under the SROC curve of SUVmax and ADC are 0.95 (95% CI, 0.92–0.96) and 0.91 (95% CI, 0.88–0.93), respectively, as shown in **Figure 5**.

**Table 3:** Summary of the diagnostic performance characteristics of SUVmax and ADC value in differentiating benign and malignant ovarian or adnexal masses.

Parameter	SUVmax		ADC value	
	Estimate	95% CI	Estimate	95% CI
Sensitivity	0.88	0.81–0.93	0.87	0.80–0.92
Specificity	0.89	0.81–0.94	0.80	0.71–0.87
PLR	7.8	4.5–13.6	4.4	2.9–6.8
NLR	0.13	0.08–0.21	0.16	0.11–0.25
DOR	59	27–128	27	14–54
AUC	0.95	0.92–0.96	0.91	0.88–0.93

PLR, positive likelihood ratio; NLR, Negative likelihood ratio; DOR, Diagnostic Odds Ratio; AUC, Area under curve; SUVmax, Maximum uptake value; ADC, Apparent diffusion coefficient.

### Publication Bias

**Figure 6:**
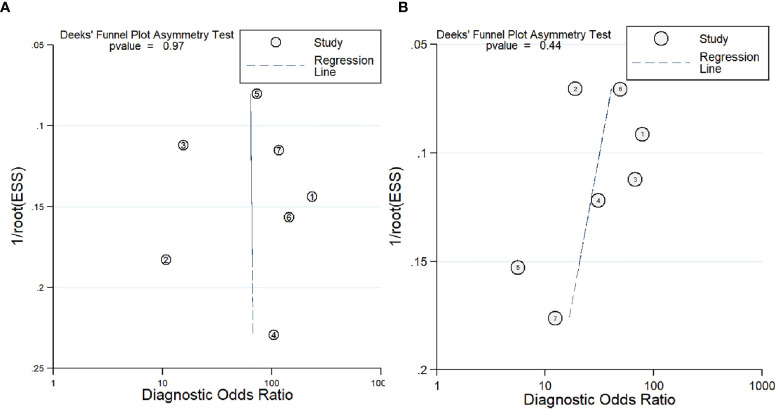
Deeks et al.’s funnel plot for publication bias for SUVmax **(A)** and ADC **(B)**.

A funnel plot for publication bias for SUVmax and ADC by Deeks et al. is shown in **Figure 6**. The *p*-values of slope coefficients were 0.97 and 0.44, respectively, which were both greater than 0.05, indicating a low possibility of publication bias.

### Exploration of Heterogeneity

To determine the source of heterogeneity between studies, univariate meta-regression analyses of studies using SUVmax and ADC to differentiate benign and malignant ovarian or adnexal masses were performed, and the results are summarized in **Table 4**. For the studies of SUVmax, the sensitivity of the cutoff value of SUVmax ≥3.0 (0.91; 95% CI: 0.86–0.95) is higher than that of SUVmax <3.0 (0.84; 95% CI: 0.78–0.90), with a *p*-value of 0.01, which may be a factor affecting heterogeneity. In terms of specificity, the sample size of the enrolled study showed heterogeneity (*p =* 0.03 <0.05). Specifically, the specificity of a sample size of more than 50 patients is higher than that of a study of 50 or less, being 0.93 (95% CI: 0.81–0.97) and 0.85 (95% CI: 0.78–0.99), respectively. The type of study design (prospective or retrospective), the vendor of PET/CT, interval time between FDG administration and scanning, FDG dose and the mean age of the enrolled patients were not factors influencing the heterogeneity between studies (all *p*-values were greater than 0.05). As for ADC, the sensitivity of the number of masses enrolled studies greater than 100 was higher than that of a study of 100 or less than, which were 0.91 (95% CI: 0.88–0.95) and 0.82 (95% CI: 0.75–0.88), respectively. Moreover, the sensitivity of the study with 6 mm (0.84; 95% CI: 0.78–0.95) as the scanning slice thickness is lower than that of the study with the scanning slice thickness of 5 mm (0.89; 95% CI: 0.80–0.95). Studies performed with a maximal *b* value of 1,000 s/mm^2^ showed lower pooled specificity (0.79; 95% CI: 0.70–0.89) compared with 800 s/mm^2^ (0.84; 95% CI: 0.68–0.95). Yet the vendor of MRI, the number of imaging planes, etc. are not factors that affect the heterogeneity between studies (*p >*0.05).

**Table 4:** The results of meta-regression analysis of SUVmax and ADC to differentiate benign and malignant ovarian or adnexal tumors.

Parameter	Category	No. of studies	Sensitivity	*p*-value	Specificity	*p*-value
SUVmax						
Design	Prospective	3	0.88 [0.78–0.98]	0.19	0.92 [0.82–1.00]	0.67
	Retrospective	3	0.92 [0.84–1.00]		0.89 [0.82–0.96]	
Mean age	≥56	3	0.92 [0.88–0.96]	0.07	0.92 [0.84–0.98]	0.96
	<56	3	0.92 [0.88–0.96]		0.91 [0.84–0.99]	
Sample	>50	4	0.88 [0.82–0.95]	0.15	0.93 [0.81–0.97]	**0.03**
	≤50	3	0.88 [0.78–0.98]		0.85 [0.78–0.99]	
Vendor	Just GE	3	0.94 [0.90–0.98]	0.33	0.85 [0.76–0.94]	0.10
	With Siemens	3	0.82 [0.76–0.89]		0.85 [0.76–0.94]	
Cutoff value	≥3.0	4	0.91 [0.86–0.95]	**0.01**	0.90 [0.82–0.99]	0.51
	<3.0	3	0.84 [0.78–0.90]		0.87 [0.78–0.97]	
Time between FDG administration and scanning	≥60 min	3	0.94 [0.88–0.99]	0.72	0.89 [0.80–0.99]	0.43
	<60 min	4	0.85 [0.78–0.91]		0.88 [0.78–0.97]	
FDG dose	≥4.0 MBq/kg	3	0.88 [0.78–0.97]	0.15	0.89 [0.77–1.00]	0.55
	<4.0 MBq/kg	3	0.86 [0.79–0.93]		0.91 [0.81–1.00]	
ADC						
China	yes	4	0.90 [0.85–0.95]	0.29	0.80 [0.69–0.90]	0.19
	no	3	0.82 [0.72–0.92]		0.81 [0.68–0.94]	
No. of tumors	≥100	3	0.91 [0.88–0.95]	**0.02**	0.78 [0.67–0.89]	0.08
	<100	4	0.82 [0.75–0.88]		0.82 [0.71–0.93]	
Max b value	1,000 s/mm^2^	4	0.91 [0.89–0.94]	0.05	0.79 [0.70–0.89]	**0.04**
	800 s/mm^2^	3	0.77 [0.70–0.84]		0.84 [0.68–0.95]	
No. of imaging planes	3	3	0.87 [0.79–0.96]	0.07	0.82 [0.71–0.93]	0.29
	2	4	0.87 [0.80–0.94]		0.79 [0.68–0.90]	
Slice thickness	6 mm	3	0.84 [0.78–0.95]	**0.04**	0.84 [0.75–0.93]	0.16
	5 mm	4	0.89 [0.80–0.95]		0.76 [0.64–0.87]	
Vendor	GE	4	0.89 [0.84–0.95]	0.22	0.78 [0.67–0.89]	0.07
	Siemens	3	0.83 [0.74–0.93]		0.83 [0.72–0.94]	

SUVmax, Maximum uptake value; ADC, Apparent diffusion coefficient.

p < 0.05 indicates that the comparison between groups is statistically significant and is indicated in bold.

## Discussion

Although there are many published studies on MRI and ^18^F-FDG PET/CT in differentiating benign and malignant ovarian or adjunct masses, the inconsistent positive reference criteria used in these studies lead to a large difference between the results. In order to minimize differences in the range of diagnostic parameters, the current meta-analysis included studies using SUVmax and ADC values to differentiate benign and malignant ovarian or adnexal masses to quantitatively evaluate their diagnostic performance and to make indirect comparisons. This comprehensive quantitative meta-analysis covered 1,317 patients with 1,373 masses from 14 studies with a wide range of features. It should be noted that in order to try to maintain consistency of patients enrolled, a study included a large number of patients with a history of primary tumors, so we excluded the data of patients with ovarian metastasis from the meta-analysis (19). The results of this meta-analysis showed that SUVmax and ADC values have good diagnostic performance in quantitatively differentiating benign and malignant ovarian or adnexal masses. Furthermore, the AUC in SUVmax is slightly higher than that in ADC value, being 0.95 and 0.91 respectively.

SUVmax represents the maximum standard uptake value in PET/CT scanning, which is equal to the ratio of imaging agent activity per unit volume of lesion tissue to injection dose, and is usually used as a quantitative indicator of ^18^F-FDG tracer uptake in tumor tissue. Clinically, SUVmax is usually used to identify benign and malignant tumors and indicate the degree of malignancy of tumors. A number of previous meta-analysis results showed that SUVmax is of great value in the staging, prognosis evaluation and monitoring of treatment response of various malignant tumors, such as breast cancer, cervical cancer and lung cancer (28–33). Moreover, a few meta-analyses results showed that SUVmax was correlated with Ki-67 index (34, 35). Our meta-analysis included 7 studies with 561 patients using SUVmax to quantitatively differentiate benign and malignant ovarian or adnexal tumors, and the results also showed good diagnostic accuracy.

ADC is used as a parameter to describe the diffusion speed and range of different water molecules in DWI-MRI, which was first applied to the central nervous system and has been recognized as an indispensable technology in many radiology fields presently (36). Compared with normal tissue or benign lesions, malignant neoplasms are usually composed of multicellular tissue and have limited diffusion in areas of high cell density, thus presenting with reduced diffusion of water molecules and a reduced apparent diffusion coefficient (37). ADC has been proven to have good diagnostic performance in the identification of benign and malignant lesions such as the brain, thyroid, pancreas, and uterus (38–42). In addition, ADC has also been confirmed in other studies to better predict the response evaluation of liver cancer, nasopharyngeal cancer, colon cancer with liver metastasis after treatment (43–45). Whether the ADC value can better distinguish benign and malignant ovarian or appendages is different in some previous studies (46, 47). However, the results of our study based on a total of 799 masses in 756 patients showed that the sensitivity and specificity of ADC values in the differentiation of ovarian or adnexal benign and malignant masses were 0.87 and 0.80, respectively, with an AUC of 0.91, indicating that quantitative ADC value is an useful diagnostic parameter for it.

The present meta-analysis shows heterogeneity in the pooled sensitivity and specificity of studies for SUVmax and ADC. The results of meta regression analysis showed that the sample size of the enrolled studies and the cutoff value of SUVmax were the factors for the heterogeneity between studies using SUVmax to evaluate benign and malignant ovarian or adnexal masses. To be specific, studies with cutoff values greater than or equal to 3.0 have a higher sensitivity to detect malignant lesions, which is not difficult to explain due to high metabolism of malignant tumors, thus there will be carbohydrate imaging agent aggregation in the lesions, resulting in higher detection of malignant lesions. Studies with a sample size of less than 50 patients showed lower specificity, which may be related to the fact that a small sample size is more likely to lead to higher false negatives. Moreover, prospective studies have higher specificity and lower sensitivity than retrospective studies, and studies conducted by Siemens has higher sensitivity than that conducted by GE only and has same specificity, but none of them are factors that cause heterogeneity (*p >*0.05). For the study of ADC, study with larger number of ovarian or adnexal masses (≥100) has a higher pooled sensitivity, which is also related to the fact that large sample sizes usually lead to smaller false negative results. The results of meta regression analysis also show that the sensitivity of the larger slice thickness is lower, which is related to the fact that smaller lesions are easily missed when the slice thickness is larger, leading to higher false negatives. Moreover, study with the maximum b value of 1,000 s/mm^2^ and performed with GE have higher sensitivity than study with the maximum *b* value of 1,000 s/mm^2^ and performed with Siemens, but they are not factors that affects the pooled sensitivity for ADC. In terms of the pooled specificity, the specificity of the study with the maximum *b* value of 1,000 s/mm^2^ is lower than that of the study with the maximum *b* value of 800 s/mm^2^, since a larger b value improves the contrast of the image while also reducing its signal-to-noise ratio. Therefore, in order to ensure a good contrast-to-noise ratio and signal-to-noise ratio, the normalized *b* value should be used in future studies and avoid using a too large *b* value to calculate the ADC (48). Moreover, the results of meta-regression showed that the scanning slice thickness was 6 mm, the image acquisition using three planes of axial, sagittal, and coronal, and the study performed by Siemens showed higher specificity, but there was no statistical difference.

Previous studies have shown that PET/CT and MRI have good diagnostic performance in the identification of benign and malignant ovarian or adnexal masses (9, 47). However, the evaluation method of these studies is a qualitative method based on MRI sequence and PET/CT metabolic parameters, which is particularly vulnerable to the subjective or bias of the researcher. The current meta-analysis results provide estimates for the diagnostic performance of quantitative SUVmax and ADC values in predicting benign and malignant ovarian or adnexal tumors, and our study indicates that quantitative both SUVmax and ADC values are useful diagnostic parameters for differentiating malignant and benign ovarian masses.

The main limitation of the current meta-analysis is that the number of eligible studies is relatively limited, while some published related studies did not specify the cutoff value of SUVmax or ADC values for positive explanations, and some studies cannot obtain TP, FP, FN, and TN values to calculate sensitivity and specificity. Secondly, the heterogeneity in the evaluation of diagnostic accuracy in both SUVmax and ADC studies also limits the quality of this meta-analysis. Finally, the indirect comparison of the diagnostic performance of SUVmax and ADC in differentiating benign and malignant ovarian or adnexal masses is also one of the limitations of the study, so prospective comparative studies are needed in future work. However, the diagnostic performance of quantitative SUVmax and ADC values of the study in differentiating benign and malignant ovarian or adnexal masses provides a reference for clinical practice and avoids the subjective interpretation of results.

In conclusion, quantitative SUVmax and ADC values showed to have high diagnostic performance in differentiating benign and malignant ovarian or adnexal masses, and the diagnostic accuracy of quantitative SUVmax is higher, both of which can be used as useful diagnostic parameters for the diagnosis of ovarian cancer. Large-scale sample size and high-quality trials to evaluate and verify the clinical value of quantitative SUVmax and ADC values in the diagnosis of ovarian cancer are needed in future work.

## Data Availability Statement

The original contributions presented in the study are included in the article/**Supplementary Material**. Further inquiries can be directed to the corresponding authors.

## Author Contributions

XH and ZL conceptualized the study, revised the manuscript and supervised the study. CZ and GW conceptualized the study, drafted the manuscript and made the figures. XH, JC and PW collected the literature and revised the manuscript. All authors contributed to the article and approved the submitted version.

## Funding

This study was funded by the National Natural Science Foundation of the Peoples Republic of China, NSFC (grant numbers: 81571712), and Qiankehe platform talents[2017] (grant numbers: 5733-035). None declared from all other authors.

## Conflict of Interest

Author GW was employed by Jiangsu Yuanben Biotechnology Co., Ltd.

The remaining authors declare that the research was conducted in the absence of any commercial or financial relationships that could be construed as a potential conflict of interest.

## Publisher’s Note

All claims expressed in this article are solely those of the authors and do not necessarily represent those of their affiliated organizations, or those of the publisher, the editors and the reviewers. Any product that may be evaluated in this article, or claim that may be made by its manufacturer, is not guaranteed or endorsed by the publisher.
