# Supplementary material for: The Diagnostic Performance of Maximum Uptake Value and Apparent Diffusion Coefficient in Differentiating Benign and Malignant Ovarian or Adnexal Masses: A Meta-Analysis
Source: Front Oncol. 2022 Feb 9;12:840433. doi: 10.3389/fonc.2022.840433 (PMC8864062; doi:10.3389/fonc.2022.840433)
Supplement: Supplementary file 1 [file Table_1.doc]

| Study | Vendor | STmin（mm） | FDG dose | Time between FDG  administration  and scanning (min) | CT technique | TP | FP | FN | TN |
| --- | --- | --- | --- | --- | --- | --- | --- | --- | --- |
| Castellucci P /2007 /Italy(14) | GE | NR | 5.5MBq/kg | 60-90 | Non-CE,  4min/bed | 28 | 0 | 4 | 18 |
| Yamamoto Y/ 2008/Japan(15) | GE | 3.75 | 4.0MBq/kg | 50 | Non-CE;  2min/bed | 10 | 3 | 4 | 13 |
| Kitajima K/2011 /Japan(16) | GE | 3.75 | 3.33MBq/kg | 50 | Non-CE=36  CE=72,  2min/bed | 70 | 6 | 15 | 20 |
| Zytoon AA  /2012Egypt(17) | Siemens | 3.75 | 3.7 – 4.5 MBq/kg | 50 | Non-CE;  2min/bed | 87 | 0 | 7 | 4 |
| Tanizaki Y/2014 /Japan(18) | Shimadzu | 2 | 2.6MBq/kg | 50 | Non-CE | 54 | 5 | 13 | 88 |
| Lee JW/2015/  Korea(19) | GE/Siemens | - | 5.5MBq/kg | 60 | Non-CE  /CE  3min/bed | 18 | 4 | 0 | 17 |
| Takagi H/2018 /Japan(20) | Siemens | - | 185MBq | 60 | Non-CE | 39 | 5 | 2 | 30 |

**Table S1.** **The characteristics of the enrolled studies to differentiate benign and malignant ovarian or adnexal masses by quantitative SUVmax of PET/CT.**

Abbreviations: GE=General Electric；STmin=minimum slice thickness；NR= not reported; CT=computed tomography; HP=histopathological; CE=Contrast enhancement; Non-CE=None contrast enhancement; TP=true positive; FP=false positive; FN=false negative; TN=true negative.

**Table S2. T****he characteristics of the enrolled studies to differentiate benign and malignant ovarian or adnexal masses by quantitative ADC values of DWI-MRI.**

| Study | Magnet strength | vendor | coverage | No.of  imaging  planes | MST  (mm) | DWI | | TP | FP | FN | TN |
| --- | --- | --- | --- | --- | --- | --- | --- | --- | --- | --- | --- |
|  |  |  |  |  |  | IP | ST(mm) |  |  |  |  |
| Li W/2011/China (21) | 1.5T | GE | pelvic | 2 | 6 | A+S | 6 | 77 | 5 | 8 | 41 |
| Zhang P/2012/China (22) | 1.5T | GE | pelvic | 2 | 5 | A+C+S | 5 | 85 | 43 | 7 | 67 |
| Fan X/2015/China (23) | 3.0T | GE | ABD+pelvic | 3 | 5 | A+C+S | 5 | 54 | 5 | 4 | 25 |
| Zhang H/2019 /China(24) | 1.5T | Siemens | pelvic | 2 | 5 | A+C+S | 5 | 51 | 3 | 11 | 20 |
| Türkoğlu S/ 2020/Turkey(25) | 1.5T | Siemens | pelvic | 3 | 5 | A | 6 | 15 | 5 | 8 | 15 |
| Mansour S/2015/Egypt (26) | MRI  (1.5T) | Siemens | pelvic | 3 | 6 | A+C+S | 6 | 148 | 12 | 15 | 60 |
| Takeuchi M/2010/Japan(27) | MRI  (1.5/3.0T) | GE | pelvic | 2 | 5 | A | 5 | 31 | 2 | 10 | 8 |

MST=minimum slice thickness; ST=slice thickness; A = axial; C = coronal; DWI = diffusion-weighted imaging; MRI = magnetic resonance imaging; ABD=abdoman; NR = not reported; S = sagittal; TP=true positive; FP=false positive; FN=false negative; TN=true negative.
